# Diagnostic efficacy of monocyte distribution width to high-sensitivity C-reactive protein ratio in differentiating pathogen types in community-acquired pneumonia: a retrospective cohort study

**DOI:** 10.1515/med-2026-1424

**Published:** 2026-05-11

**Authors:** Rong Yu, Hua Tang

**Affiliations:** Clinical Laboratory, The Second Hospital of Hunan University of Chinese Medicine, Changsha City, Hunan, China; Department of Medical Genetic, The Maternal and Child Health Hospital of Hunan Province, Changsha City, Hunan, China

**Keywords:** community-acquired pneumonia, monocyte distribution width, high-sensitivity C-reactive protein, ratio biomarker, diagnostic test

## Abstract

**Objectives:**

Community-acquired pneumonia (CAP) remains a diagnostic challenge in emergency departments, where rapid and accurate pathogen differentiation is essential for appropriate antimicrobial therapy and stewardship. Current biomarkers demonstrate limited accuracy in distinguishing bacterial from viral and atypical pathogens, necessitating novel diagnostic approaches. This study aimed to assess the diagnostic accuracy of the monocyte distribution width to high-sensitivity C-reactive protein ratio (MDW/hs-CRP) for differentiating bacterial, viral, atypical, and mixed community-acquired pneumonia (CAP) pathogens.

**Methods:**

This retrospective cohort study enrolled 486 adult patients with radiologically confirmed CAP in an emergency department (Jan 2023–Dec 2024). Pathogens were identified using comprehensive microbiological testing. MDW was measured via automated hematology analyzer (Beckman Coulter DxH900); hs-CRP via standard assays. The primary outcome compared the MDW/hs-CRP ratio’s diagnostic accuracy to individual biomarkers using ROC analysis (DeLong test), Youden index (cutoff), decision curve analysis (DCA), and subgroup analyses.

**Results:**

Pathogen distribution: 198 (40.7 %) bacterial, 142 (29.2 %) viral, 89 (18.3 %) atypical, 57 (11.7 %) mixed. For distinguishing bacterial from viral CAP, the MDW/hs-CRP ratio significantly outperformed MDW alone (AUC 0.752) and hs-CRP alone (AUC 0.798), achieving an AUC of 0.893 (95 % CI: 0.865–0.921; p<0.001). The optimal cutoff (0.185) yielded 85.4 % sensitivity and 82.7 % specificity. Performance remained consistent in subgroups. DCA showed clinical utility across threshold probabilities (15–65 % net benefit).

**Conclusions:**

The MDW/hs-CRP ratio shows promise as a rapid tool for pathogen differentiation in CAP, potentially aiding early antimicrobial decisions in emergency care. Multicenter validation is needed.

## Introduction

Community-acquired pneumonia (CAP) remains a leading cause of morbidity and mortality worldwide, with an estimated annual incidence of 5–11 per 1,000 adults and mortality rates ranging from 5 to 15 % in hospitalized patients [1]. The accurate and timely differentiation of bacterial from viral and atypical pathogens represents a critical challenge in emergency medicine, with profound implications for antimicrobial stewardship and patient outcomes [2]. Current diagnostic approaches, including clinical scoring systems and traditional biomarkers, demonstrate suboptimal accuracy in distinguishing pathogen types, leading to inappropriate antibiotic use in up to 40 % of cases [3].

Monocyte distribution width (MDW) has emerged as a novel biomarker reflecting the morphological heterogeneity of circulating monocytes in response to systemic inflammation [4]. As a measure of monocytic anisocytosis detected through volume, conductivity, and scatter (VCS) technology, MDW increases during the early phases of immune activation, with differential patterns observed between bacterial and viral infections [5]. The FDA approval of MDW in 2019 as an adjunct sepsis biomarker has catalyzed research into its broader diagnostic applications [6]. Recent meta-analyses have reported pooled sensitivities of 0.69–0.79 and specificities of 0.57–0.86 for sepsis detection, with performance varying by clinical setting and reference standard [7].

High-sensitivity C-reactive protein (hs-CRP), an established acute-phase reactant synthesized by hepatocytes in response to proinflammatory cytokines, exhibits well-characterized kinetics in infectious diseases [8]. While hs-CRP demonstrates reasonable sensitivity for bacterial infections, its specificity remains limited due to elevation in diverse inflammatory conditions [9]. Studies in CAP have shown that CRP levels above 40 mg/L have sensitivity of 70–73 % and specificity of 65–90 % for bacterial pneumonia, though substantial overlap exists between pathogen groups [10].

The biological rationale for combining MDW and hs-CRP into a single ratio metric stems from their complementary immunological mechanisms and temporal dynamics. MDW reflects early innate immune activation through monocyte morphological changes, while hs-CRP represents the downstream hepatic response to inflammatory mediators [11]. Mathematical modeling suggests that ratio-based biomarkers may amplify pathogen-specific signal differences while normalizing for individual baseline variation [12]. This approach parallels the successful application of neutrophil-to-lymphocyte ratio (NLR) and other composite inflammatory indices in infectious disease diagnostics [13].

Despite growing interest in composite biomarkers, no studies have systematically evaluated the MDW/hs-CRP ratio for CAP pathogen differentiation. This retrospective study represents the first comprehensive evaluation of this novel ratio as a diagnostic tool for pathogen differentiation in CAP. We hypothesized that the MDW/hs-CRP ratio would demonstrate superior diagnostic accuracy compared to individual markers, providing a rapid, cost-effective tool for emergency antimicrobial decision-making that could be readily integrated into routine laboratory workflows without requiring specialized testing platforms.

## Methods

### Study design and participants

We conducted a retrospective cohort study at The Second Hospital of Hunan University of Chinese Medicine emergency department between January 2023 and December 2024. Consecutive adult patients (≥18 years) presenting to the emergency department with suspected CAP were screened for eligibility through review of electronic medical records. Inclusion criteria comprised: (1) clinical presentation consistent with lower respiratory tract infection (new or worsening cough, sputum production, dyspnea, or pleuritic chest pain); (2) radiographic evidence of new pulmonary infiltrate on chest radiograph or computed tomography; (3) symptom onset within 14 days; and (4) blood sampling within 6 h of presentation. Exclusion criteria included: (1) hospitalization within the preceding 30 days; (2) residence in long-term care facilities; (3) immunosuppression (HIV with CD4 <200 cells/μL, solid organ or hematopoietic transplantation, chemotherapy within 30 days, or prednisone >20 mg/day for >14 days); (4) active hematological malignancy; (5) prior antibiotic therapy >48 h; (6) pregnancy or lactation; and (7) incomplete medical records or missing biomarker data. Sample size calculation targeted detection of an AUC difference of 0.10 between the MDW/hs-CRP ratio and individual biomarkers. Assuming a baseline AUC of 0.75 for single markers and equal proportions of bacterial vs. non-bacterial cases, 420 evaluable patients were required to achieve 80 % statistical power at a two-sided significance level of 0.05. To account for approximately 15 % exclusion due to incomplete records, we targeted enrollment of 495 patients.

### Biomarker measurements and laboratory procedures

Venous blood samples were collected in K3-EDTA tubes for MDW analysis and serum separator tubes for hs-CRP measurement at the time of initial emergency department evaluation, prior to antibiotic administration when feasible. MDW was measured using the UniCel DxH900 hematology analyzer (Beckman Coulter, Miami, FL, USA) within 2 h of collection. The analyzer employs VCS technology to identify monocytes based on individual cell volume, high-frequency conductivity, and laser light scatter patterns, calculating MDW as the standard deviation of monocyte volume distribution expressed in femtoliters (fL). Quality control procedures included daily calibration with manufacturer-provided standards and participation in external quality assessment programs. The coefficient of variation for MDW measurements was 3.2 % at low levels (18 units) and 2.8 % at high levels (28 units). High-sensitivity CRP was measured using a latex-enhanced immunoturbidimetric assay on the Roche Cobas c702 analyzer (Roche Diagnostics, Basel, Switzerland) with analytical sensitivity of 0.15 mg/L and measurement range of 0.15–350 mg/L, expressed in milligrams per liter (mg/L). Samples exceeding the upper limit were diluted and reanalyzed. Inter-assay coefficients of variation were 2.1 % at 10 mg/L and 1.8 % at 80 mg/L. The MDW/hs-CRP ratio was calculated as MDW (fL) divided by hs-CRP (mg/L), yielding a dimensionless ratio value. Additional laboratory assessments included complete blood count with differential, procalcitonin (PCT) by electrochemiluminescence immunoassay, comprehensive metabolic panel, and calculation of NLR, platelet-to-lymphocyte ratio (PLR), and monocyte-to-lymphocyte ratio (MLR). These supplementary markers served as comparators in sensitivity analyses but were not components of the primary diagnostic algorithm.

### Pathogen classification and microbiological testing

Etiological classification followed a hierarchical approach integrating multiple diagnostic modalities. Bacterial CAP was defined by: (1) positive blood cultures with recognized respiratory pathogens (*Streptococcus pneumoniae*, *Haemophilus influenzae*, *Staphylococcus aureus*, *Klebsiella pneumoniae*, *Pseudomonas aeruginosa*, *Escherichia coli*, or other gram-negative bacilli); (2) positive sputum or bronchoalveolar lavage cultures with >10^5^ CFU/mL of pathogenic bacteria and compatible Gram stain; (3) positive urinary antigen tests for *S. pneumoniae* or *Legionella pneumophila* serogroup 1; or (4) positive pneumococcal PCR from normally sterile sites. Among the 198 bacterial CAP cases, 89 (44.9 %) were culture-confirmed, 67 (33.8 %) diagnosed by urinary antigen alone, and 42 (21.2 %) by PCR methods. The predominant bacterial pathogens were *S. pneumoniae* (n=112, 56.6 %), *H. influenzae* (n=34, 17.2 %), *S. aureus* (n=28, 14.1 %), *K. pneumoniae* (n=16, 8.1 %), and others (n=8, 4.0 %).

Viral CAP required detection of respiratory viruses by multiplex PCR panel (FilmArray Respiratory Panel 2.1, BioFire Diagnostics) including influenza A/B, respiratory syncytial virus, parainfluenza viruses, human metapneumovirus, rhinovirus/enterovirus, coronaviruses (including SARS-CoV-2), and adenovirus, in the absence of bacterial co-pathogens. Viral pathogens identified included influenza A (n=52, 36.6 %), influenza B (n=28, 19.7 %), respiratory syncytial virus (n=24, 16.9 %), SARS-CoV-2 (n=18, 12.7 %), rhinovirus/enterovirus (n=12, 8.5 %), and others (n=8, 5.6 %). Seasonal distribution showed 68 % of viral cases occurring between November 2023 and March 2024.

Atypical CAP was diagnosed through: (1) *Mycoplasma pneumoniae* or *Chlamydophila pneumoniae* PCR positivity; (2) four-fold rise in specific antibody titers between acute and convalescent sera; or (3) positive Legionella culture or PCR from respiratory specimens. Atypical pathogens included *M. pneumoniae* (n=62, 69.7 %), *C. pneumoniae* (n=18, 20.2 %), and *L. pneumophila* (n=9, 10.1 %). Mixed infections were defined as concurrent detection of pathogens from two or more categories. Patients without definitive microbiological diagnosis despite comprehensive testing (n=94, 16.2 % of screened patients with complete biomarker data) were excluded from primary analyses but included in prespecified sensitivity analyses as “undetermined etiology.”

Two independent clinical microbiologists blinded to biomarker results reviewed all microbiological data for final pathogen classification. Discordant cases were adjudicated by a third reviewer, with consensus required for final categorization. Inter-rater agreement, assessed by Cohen’s kappa, was 0.89 (95 % CI: 0.85–0.93).

### Clinical data collection and outcome assessment

Standardized data extraction forms captured demographic characteristics, comorbidities, symptom duration, vital signs, and physical examination findings at presentation from electronic medical records. Disease severity was assessed using the Pneumonia Severity Index (PSI) and CURB-65 score, calculated from admission data. The Charlson Comorbidity Index quantified comorbidity burden. Time from symptom onset to presentation was recorded in days, with verification through electronic health records when available. Primary outcomes included diagnostic accuracy metrics (sensitivity, specificity, positive and negative predictive values, likelihood ratios) for the MDW/hs-CRP ratio in differentiating: (1) bacterial vs. viral CAP; (2) bacterial/atypical vs. viral CAP; and (3) any bacterial involvement vs. purely viral CAP. Secondary outcomes encompassed length of hospital stay, intensive care unit admission, mechanical ventilation requirement, 30-day mortality, and antibiotic duration. Clinical outcomes were extracted from electronic health records with manual verification of ambiguous entries.

### Statistical analysis

Baseline characteristics were summarized using means with standard deviations or medians with interquartile ranges for continuous variables, and frequencies with percentages for categorical variables. Between-group comparisons employed Student’s t-test or Mann-Whitney U test for continuous data and chi-square or Fisher’s exact test for categorical data, as appropriate. Receiver operating characteristic (ROC) curves were constructed for the MDW/hs-CRP ratio and individual biomarkers, with areas under the curve (AUC) calculated using the trapezoidal rule. The DeLong method compared AUCs between markers, with Bonferroni correction for multiple comparisons. Optimal cutoff points were determined using the Youden index (maximizing sensitivity + specificity − 1), with 95 % confidence intervals derived from 2000 bootstrap replicates. Diagnostic performance metrics were calculated at both optimal and prespecified clinically relevant thresholds (95 % sensitivity threshold and 95 % specificity threshold). Confusion matrices were constructed at the optimal cutoff to visualize classification performance.

Multivariable logistic regression models evaluated the independent association of the MDW/hs-CRP ratio with bacterial CAP, adjusting for age, sex, comorbidities, disease severity, and symptom duration. Model calibration was assessed using the Hosmer–Lemeshow test and calibration plots. Discrimination was quantified by the concordance index. Multicollinearity was evaluated through variance inflation factors, with values >5 prompting variable reconsideration.

Decision curve analysis (DCA) quantified the net benefit of using the MDW/hs-CRP ratio across a range of threshold probabilities (0–100 %), comparing strategies of treating all patients, treating none, and biomarker-guided treatment. Net benefit was calculated as (true positives/n) − (false positives/n) × (threshold probability/(1−threshold probability)), where n represents the total sample size. The clinical utility of the ratio was assessed by the range of threshold probabilities yielding positive net benefit compared to default strategies.

Prespecified subgroup analyses examined diagnostic performance stratified by: (1) age (<65 vs. ≥65 years); (2) disease severity (PSI class I–III vs. IV–V); (3) comorbidity burden (Charlson index 0–1 vs. ≥2); (4) time from symptom onset (<3 vs. ≥3 days); and (5) prior antibiotic exposure (<48 h vs. none). Interaction terms in logistic models tested for differential performance across subgroups.

Sensitivity analyses included: (1) inclusion of undetermined etiology cases; (2) alternative ratio calculations (log-transformed, standardized); (3) exclusion of mixed infections; (4) restriction to culture-confirmed cases; (5) imputation of missing biomarker values using multiple imputation by chained equations; and (6) application of different cutoff selection methods (closest-to-corner, cost-benefit weighted).

All analyses were performed using R version 4.3.0 (R Foundation for Statistical Computing, Vienna, Austria) with packages pROC for ROC analyses, rms for regression modeling, and dcurves for decision curve analysis. Statistical significance was defined as two-tailed p<0.05 unless otherwise specified. The study protocol received approval from the Institutional Review Board of The Second Hospital of Hunan University of Chinese Medicine (Approval Number: 2023-156), with informed consent requirements waived given the retrospective design.

## Results

### Study population and baseline characteristics

**Figure 1:**
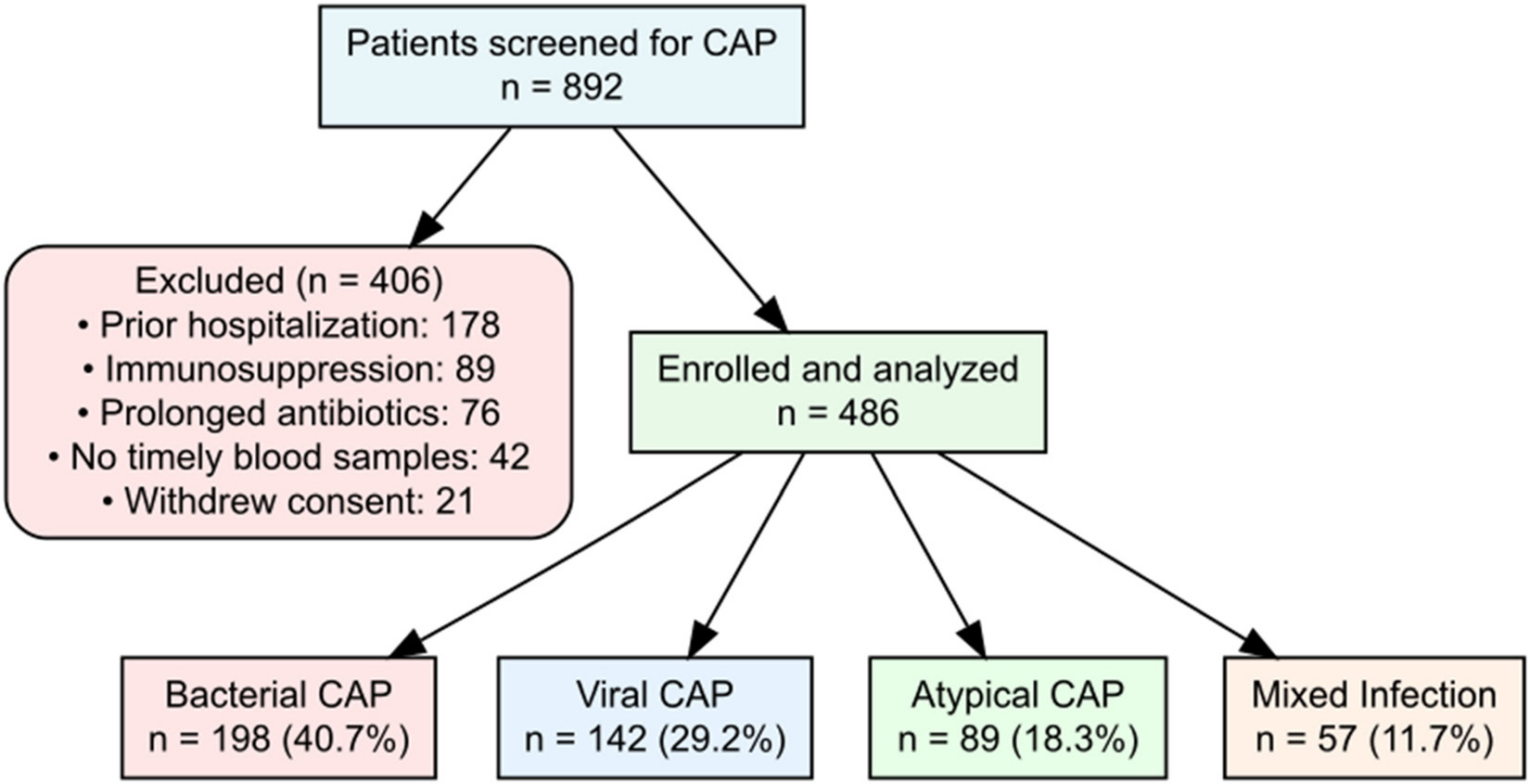
Study enrollment flowchart showing screening of 892 patients, exclusions (n=406), and final classification into bacterial (n=198), viral (n=142), atypical (n=89), and mixed infection (n=57) groups.

**Figure 2:**
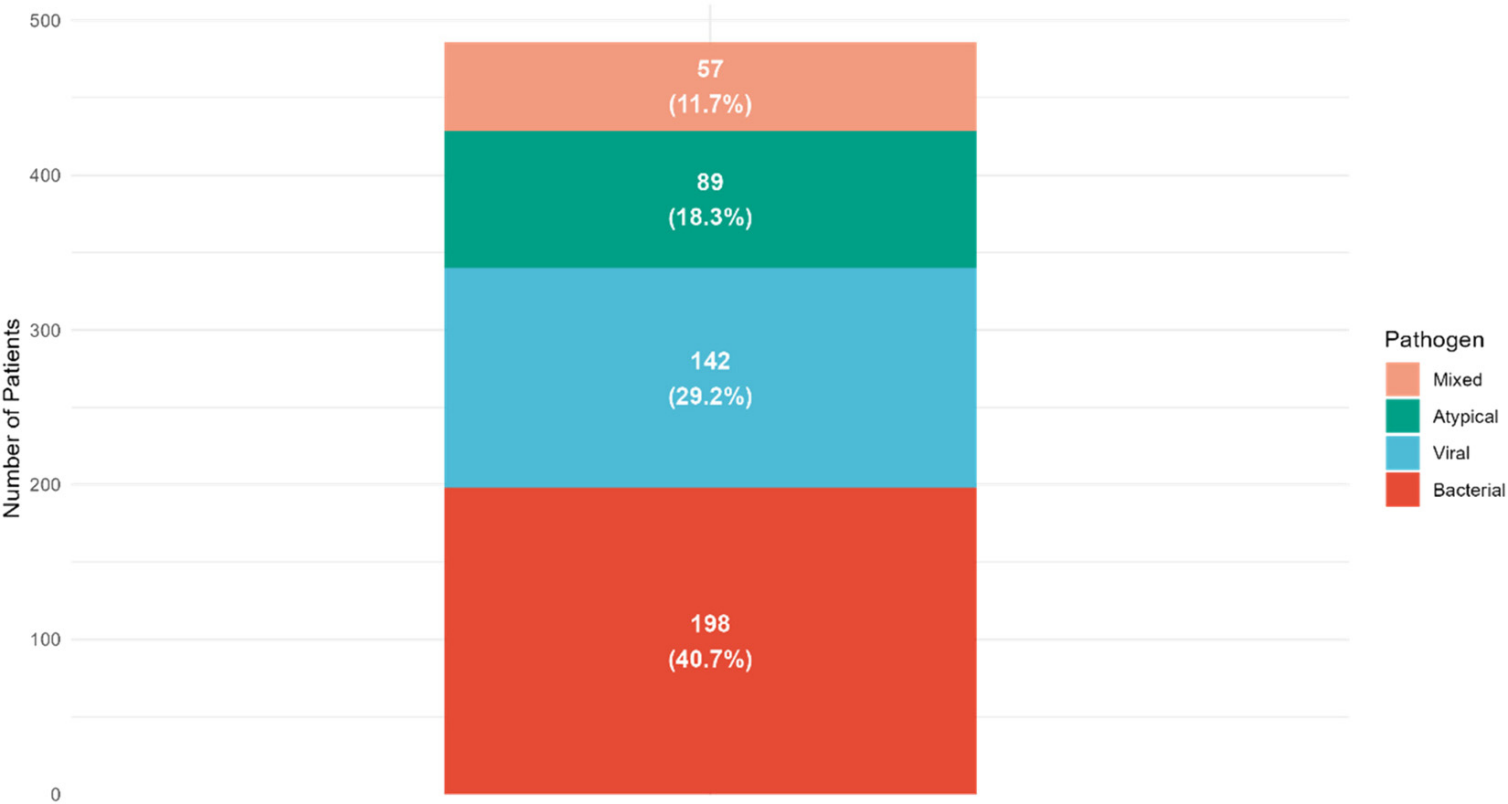
Distribution of pathogen types in the study cohort. Bacterial infections predominated (198 cases, 40.7 %), followed by viral (142 cases, 29.2 %), atypical (89 cases, 18.3 %), and mixed infections (57 cases, 11.7 %). This distribution reflects typical CAP epidemiology in emergency department settings and provides the foundation for subsequent biomarker performance analysis.

During the 24-month enrollment period, 892 patients with suspected CAP were screened through retrospective chart review, of whom 486 met inclusion criteria and had complete study data available (Figure 1). Exclusions included prior hospitalization (n=178), immunosuppression (n=89), prolonged antibiotic therapy (n=76), inability to obtain timely blood samples or missing biomarker data (n=42), and incomplete medical records (n=21). The final cohort comprised 198 (40.7 %) bacterial CAP, 142 (29.2 %) viral CAP, 89 (18.3 %) atypical CAP, and 57 (11.7 %) mixed infection cases (Figure 2).

Marked differences in baseline characteristics emerged across pathogen groups (Table 1). Compared to viral CAP patients, those with bacterial CAP were considerably older (median age 68 vs. 52 years, p<0.001), carried a substantially higher comorbidity burden (Charlson comorbidity index ≥2: 54.5% vs. 28.2 %, p<0.001), and manifested more severe disease at presentation (PSI risk class IV–V: 48.5% vs. 19.0 %, p<0.001). Bacterial CAP patients reported significantly shorter pre-presentation symptom duration (median 3 vs. 5 days, p=0.002). Classic bacterial infection indicators were substantially more common in this group, including high-grade fever ≥38.5 °C (72.2% vs. 45.1 %, p<0.001), productive cough (81.3% vs. 52.8 %, p<0.001), and pleuritic chest pain (35.4% vs. 16.9 %, p=0.001).

**Table 1:** Baseline characteristics of study population by pathogen group.

Characteristic	Bacterial, n=198	Viral, n=142	Atypical, n=89	Mixed, n=57	p-Value
Age, years, median, IQR	68 (56–78)	52 (38–65)	45 (32–58)	61 (48–72)	<0.001
Male sex, n, %	112 (56.6)	68 (47.9)	42 (47.2)	31 (54.4)	0.342
Charlson index ≥2, n, %	108 (54.5)	40 (28.2)	18 (20.2)	28 (49.1)	<0.001
Diabetes mellitus, n, %	62 (31.3)	28 (19.7)	12 (13.5)	18 (31.6)	0.008
COPD, n, %	48 (24.2)	22 (15.5)	8 (9.0)	14 (24.6)	0.021
PSI class IV-V, n, %	96 (48.5)	27 (19.0)	11 (12.4)	22 (38.6)	<0.001
CURB-65 ≥2, n, %	89 (44.9)	31 (21.8)	14 (15.7)	24 (42.1)	<0.001
Symptom duration, days	3 (2–5)	5 (3–7)	7 (5–10)	4 (3–6)	<0.001
Fever ≥38.5 °C, n, %	143 (72.2)	64 (45.1)	52 (58.4)	38 (66.7)	<0.001
Multilobar infiltrates, n, %	74 (37.4)	28 (19.7)	12 (13.5)	21 (36.8)	<0.001

### Laboratory findings and biomarker distributions

**Figure 3:**
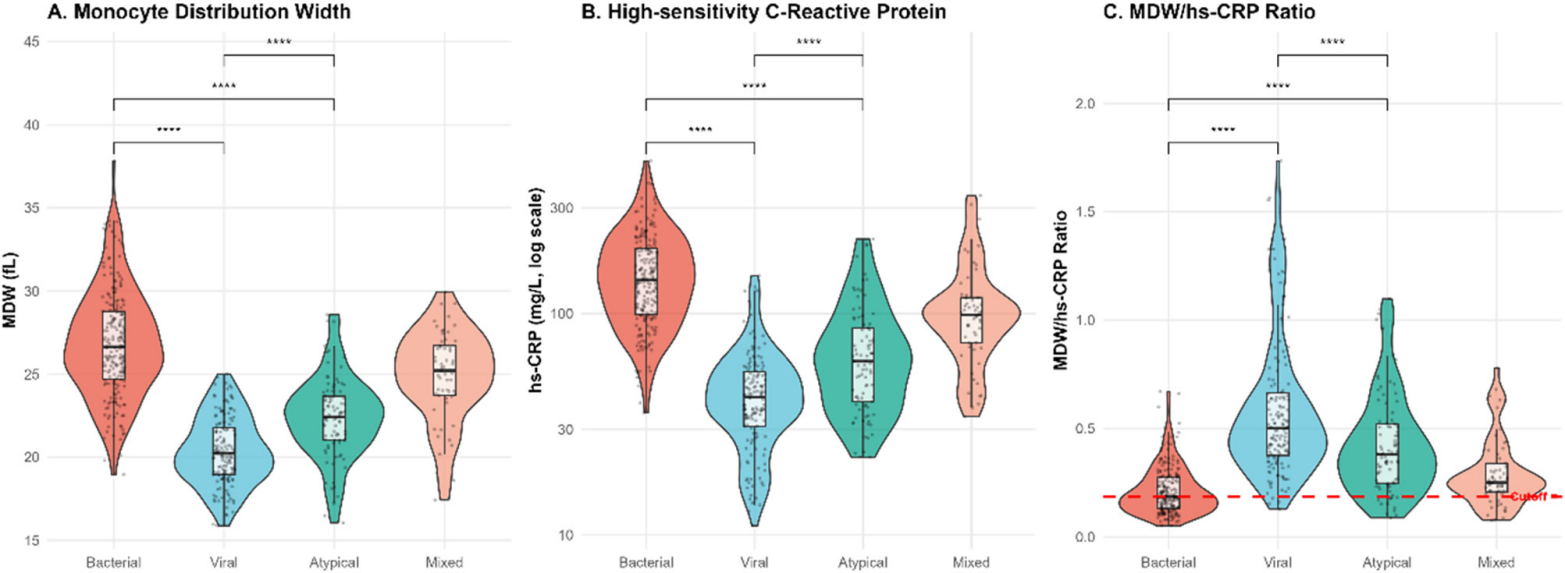
Violin plots displaying the distribution of MDW, hs-CRP, and MDW/hs-CRP ratio values across pathogen groups. The MDW/hs-CRP ratio demonstrates superior discrimination between bacterial (median 0.184, IQR 0.142–0.256) and viral infections (median 0.523, IQR 0.368–0.742) compared to individual biomarkers, with minimal distribution overlap (p<0.001). This substantial separation magnitude supports the ratio’s clinical utility for rapid pathogen-type differentiation in emergency settings.

Laboratory parameters revealed striking pathogen-specific patterns (Table 2). Bacterial CAP demonstrated markedly elevated MDW levels (median 26.8 fL) relative to viral (20.2 fL), atypical (22.4 fL), and mixed infections (24.6 fL), with all pairwise comparisons reaching statistical significance (p<0.001). High-sensitivity CRP followed a similar trend, showing dramatic elevation in bacterial cases (median 145.2 mg/L) compared to viral infections (38.6 mg/L, p<0.001); however, considerable inter-group overlap limited its discriminatory power. Most notably, the MDW/hs-CRP ratio achieved exceptional separation between bacterial (median 0.184) and viral CAP (median 0.523) (p<0.001), as visualized in Figure 3.

**Table 2:** Laboratory parameters and biomarkers by pathogen type.

Parameter	Bacterial	Viral	Atypical	Mixed	p-Value
WBC, ×10^9^/L	14.2 (9.8–18.6)	7.8 (5.9–10.2)	9.2 (6.8–11.8)	11.4 (8.2–15.1)	<0.001
Neutrophils, ×10^9^/L	11.8 (7.9–15.8)	5.2 (3.8–7.1)	6.8 (4.9–8.9)	8.9 (6.2–12.1)	<0.001
Lymphocytes, ×10^9^/L	0.9 (0.6–1.3)	1.4 (0.9–2.0)	1.6 (1.1–2.2)	1.1 (0.7–1.5)	<0.001
Monocytes, ×10^9^/L	0.8 (0.5–1.1)	0.6 (0.4–0.8)	0.7 (0.5–0.9)	0.7 (0.5–1.0)	0.042
MDW, fL	26.8 (23.4–30.2)	20.2 (18.1–22.4)	22.4 (20.1–24.8)	24.6 (21.8–27.2)	<0.001
hs-CRP, mg/L	145.2 (82.4–218.6)	38.6 (18.2–65.4)	56.8 (28.4–92.6)	98.4 (52.8–156.2)	<0.001
MDW/hs-CRP ratio	0.184 (0.142–0.256)	0.523 (0.368–0.742)	0.394 (0.286–0.548)	0.250 (0.186–0.382)	<0.001
PCT, ng/mL	2.8 (0.8–8.6)	0.2 (0.1–0.4)	0.4 (0.2–0.8)	1.2 (0.5–3.2)	<0.001
NLR	13.1 (8.2–19.4)	3.7 (2.4–5.8)	4.3 (2.9–6.2)	8.1 (5.2–12.4)	<0.001
PLR	285 (189–412)	142 (98–205)	156 (108–218)	218 (156–302)	<0.001
MLR	0.89 (0.56–1.32)	0.43 (0.28–0.62)	0.44 (0.31–0.58)	0.64 (0.45–0.92)	<0.001

Values are median (IQR). WBC, white blood cell; MDW, monocyte distribution width; hs-CRP, high-sensitivity C-reactive protein; PCT, procalcitonin; NLR, neutrophil-to-lymphocyte ratio; PLR, platelet-to-lymphocyte ratio; MLR, monocyte-to-lymphocyte ratio.

### Diagnostic performance analysis

**Figure 4:**
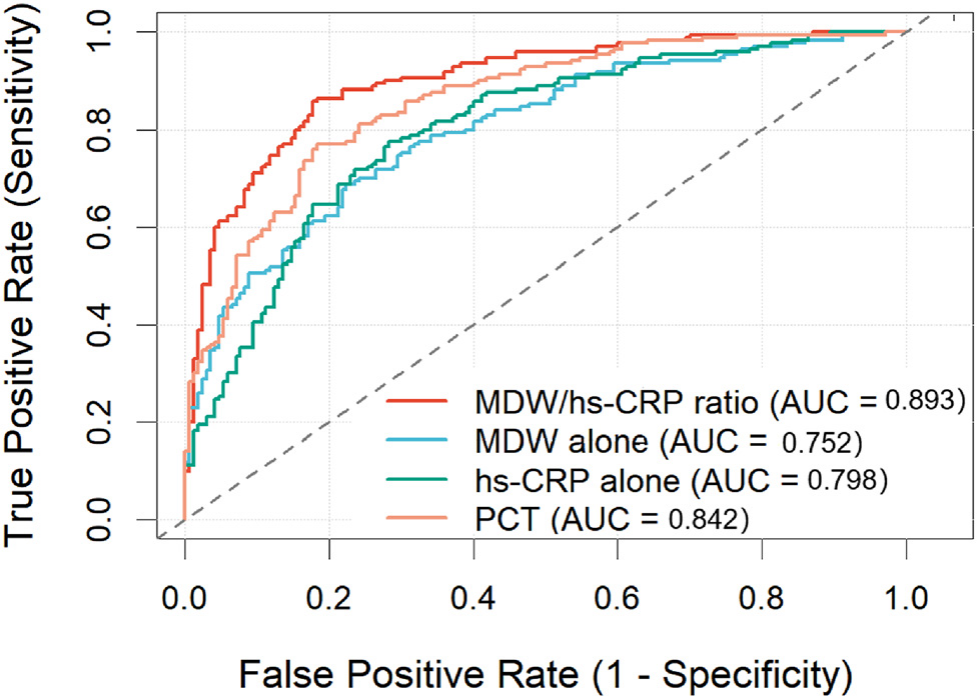
ROC curves comparing diagnostic performance of MDW/hs-CRP ratio (AUC 0.893), MDW (AUC 0.752), hs-CRP (AUC 0.798), and PCT (AUC 0.842) for bacterial vs. viral CAP differentiation.

ROC curve analysis for bacterial vs. viral CAP differentiation established clear superiority of the MDW/hs-CRP ratio (AUC 0.893, 95 % CI: 0.865–0.921) over MDW alone (AUC 0.752, 95 % CI: 0.708–0.796, p<0.001), hs-CRP alone (AUC 0.798, 95 % CI: 0.758–0.838, p<0.001), and even procalcitonin (AUC 0.842, 95 % CI: 0.806–0.878, p=0.018) (Figure 4, Table 3). At the statistically optimal cutoff value of 0.185, the ratio achieved sensitivity of 85.4 % (95 % CI: 79.8–89.9 %), specificity of 82.7 % (95 % CI: 75.5–88.4 %), positive likelihood ratio of 4.94 (95 % CI: 3.52–6.92), and negative likelihood ratio of 0.18 (95 % CI: 0.12–0.25).

**Table 3:** Diagnostic performance for bacterial vs. viral CAP differentiation.

Biomarker	AUC (95 % CI)	Cutoff	Sensitivity	Specificity	PPV	NPV	LR+	LR−
MDW/hs-CRP ratio	0.893 (0.865–0.921)	0.185	85.4 %	82.7 %	86.2 %	81.8 %	4.94	0.18
MDW alone	0.752 (0.708–0.796)	23.5 fL	72.2 %	71.8 %	76.1 %	67.5 %	2.56	0.39
hs-CRP alone	0.798 (0.758–0.838)	72 mg/L	76.8 %	73.9 %	78.4 %	72.1 %	2.94	0.31
PCT	0.842 (0.806–0.878)	0.5 ng/mL	79.3 %	78.2 %	81.8 %	75.5 %	3.64	0.26
NLR	0.826 (0.788–0.864)	7.0	77.3 %	76.8 %	80.1 %	73.6 %	3.33	0.30
MDW + hs-CRP^a^	0.908 (0.882–0.934)	–	87.9 %	84.5 %	87.9 %	84.5 %	5.67	0.14

^a^Combined model using logistic regression. PPV, positive predictive value; NPV, negative predictive value; LR+, positive likelihood ratio; LR−: negative likelihood ratio. For distinguishing bacterial/atypical from viral CAP, the MDW/hs-CRP, ratio maintained robust performance (AUC, 0.871, 95 % CI: 0.842–0.900), superior to individual markers. In the three-way classification (bacterial vs. viral vs. atypical), the ratio demonstrated moderate discriminatory ability with overall accuracy of 72.4 %.

To accommodate varying clinical priorities, we identified two alternative threshold values. When maximum sensitivity was prioritized (95 %), a cutoff of 0.265 delivered 95.0 % sensitivity paired with 68.3 % specificity – ideal for screening scenarios where missing bacterial cases carries high clinical risk. Conversely, when maximum specificity was prioritized (95 %), a cutoff of 0.128 yielded 95.2 % specificity with 68.2 % sensitivity – optimal when diagnostic certainty is required before initiating aggressive antibiotic therapy. At the optimal balanced cutoff (0.185), our confusion matrix revealed 169 true positives, 117 true negatives, 25 false positives, and 29 false negatives. This flexible threshold approach empowers clinicians to calibrate diagnostic strategy according to individual patient risk profiles and institutional antibiotic stewardship goals.

### Multivariable analysis and independent associations

**Figure 5:**
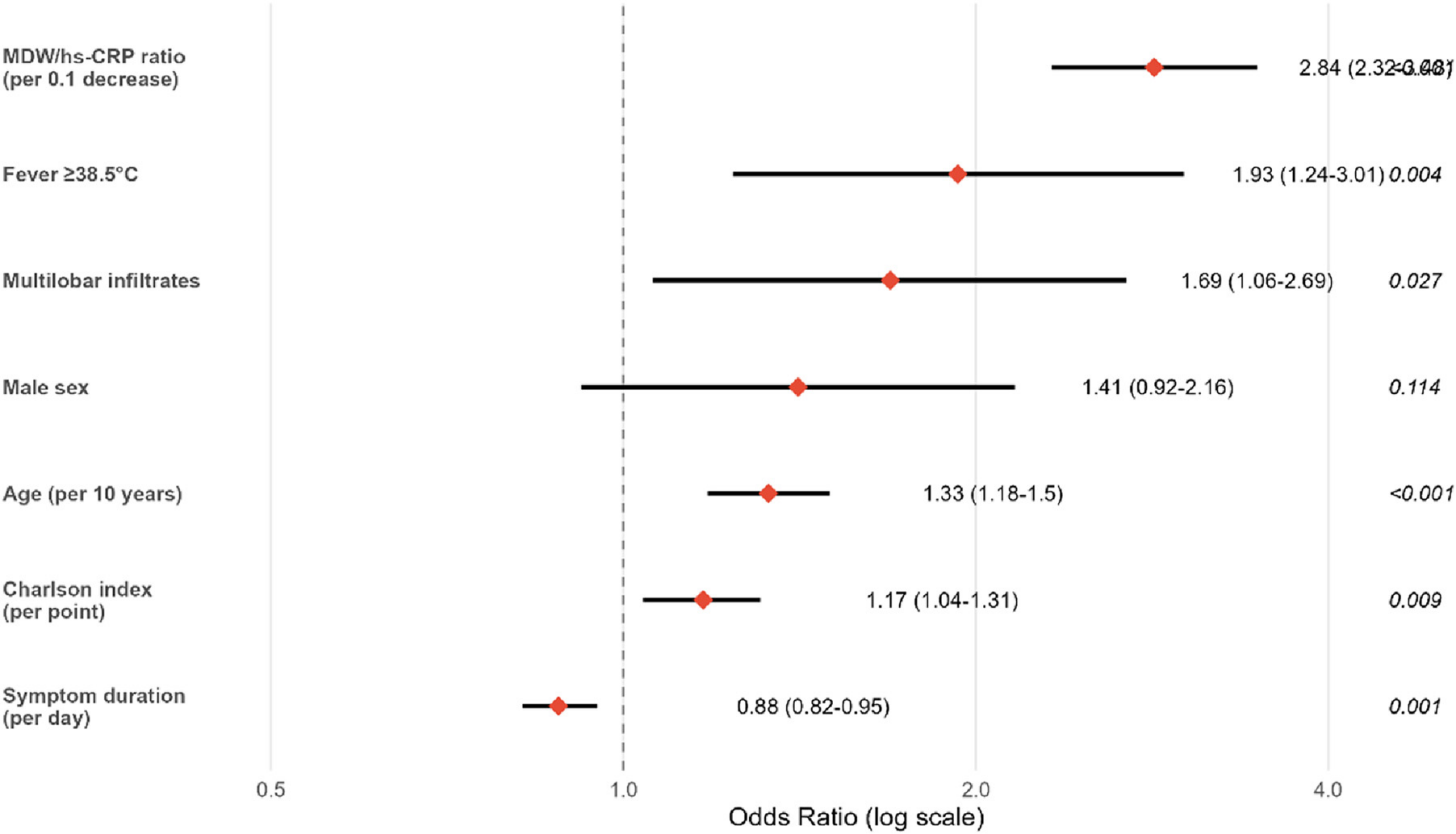
Forest plot displaying adjusted odds ratios from multivariable logistic regression analysis. The MDW/hs-CRP ratio emerges as the strongest independent predictor of bacterial CAP (OR 2.84, 95 % CI 2.12–3.80, p<0.001) after controlling for clinical factors including fever, multilobar infiltrates, age, Charlson comorbidity index, and symptom duration. This robust independent association underscores the ratio’s value beyond conventional clinical assessment, supporting its integration into diagnostic algorithms for emergency department CAP evaluation.

After rigorous adjustment for established clinical predictors, multivariable logistic regression confirmed the MDW/hs-CRP ratio as a powerful independent predictor of bacterial CAP etiology (Table 4). Specifically, each 0.1-unit decrease in the ratio conferred 2.84-fold increased odds of bacterial infection (95 % CI: 2.32–3.48, p<0.001). The final multivariable model demonstrated excellent statistical properties, including strong calibration (Hosmer–Lemeshow goodness-of-fit p=0.42) and outstanding discrimination (c-statistic 0.926), as depicted in Figure 5.

**Table 4:** Multivariable logistic regression for bacterial CAP prediction.

Variable	Coefficient	OR (95 % CI)	p-Value	VIF
MDW/hs-CRP ratio (per 0.1 decrease)	1.044	2.84 (2.32–3.48)	<0.001	1.12
Age (per 10 years)	0.285	1.33 (1.18–1.50)	<0.001	1.24
Male sex	0.342	1.41 (0.92–2.16)	0.114	1.08
Fever ≥38.5 °C	0.658	1.93 (1.24–3.01)	0.004	1.15
Symptom duration (per day)	−0.124	0.88 (0.82–0.95)	0.001	1.09
Charlson index (per point)	0.156	1.17 (1.04–1.31)	0.009	1.28
Multilobar infiltrates	0.524	1.69 (1.06–2.69)	0.027	1.11

OR, odds ratio; CI, confidence interval; VIF, variance inflation factor.

### Subgroup and sensitivity analyses

The diagnostic performance of the MDW/hs-CRP ratio proved remarkably stable across clinically relevant subgroups, with no statistically significant interactions detected (all p-interaction values >0.10). Among elderly patients (age ≥65 years), the ratio maintained an AUC of 0.882 (95 % CI: 0.841–0.923), virtually identical to performance in younger patients (AUC 0.898, p=0.62 for comparison). Robust performance persisted in high-risk subgroups including severe pneumonia (PSI class IV–V: AUC 0.876) and patients with substantial comorbidity burden (Charlson index ≥2: AUC 0.888). Notably, timing of presentation relative to symptom onset did not materially impact diagnostic accuracy (early presentation <3 days: AUC 0.896 vs. delayed presentation ≥3 days: AUC 0.885, p=0.71).

Multiple sensitivity analyses confirmed the robustness of our primary findings. Incorporating 94 additional patients with indeterminate etiology (those lacking definitive microbiological diagnosis despite exhaustive testing) yielded nearly identical results (AUC 0.878). Alternative mathematical transformations of the ratio produced comparable discriminatory power (natural log-transformed ratio: AUC 0.889). Restricting analysis exclusively to culture-confirmed cases (n=156) preserved diagnostic accuracy (AUC 0.904). Finally, multiple imputation procedures to address missing biomarker values (affecting 8.2 % of measurements) generated consistent estimates (pooled AUC 0.891 across imputed datasets).

### Decision curve analysis and clinical utility

**Figure 6:**
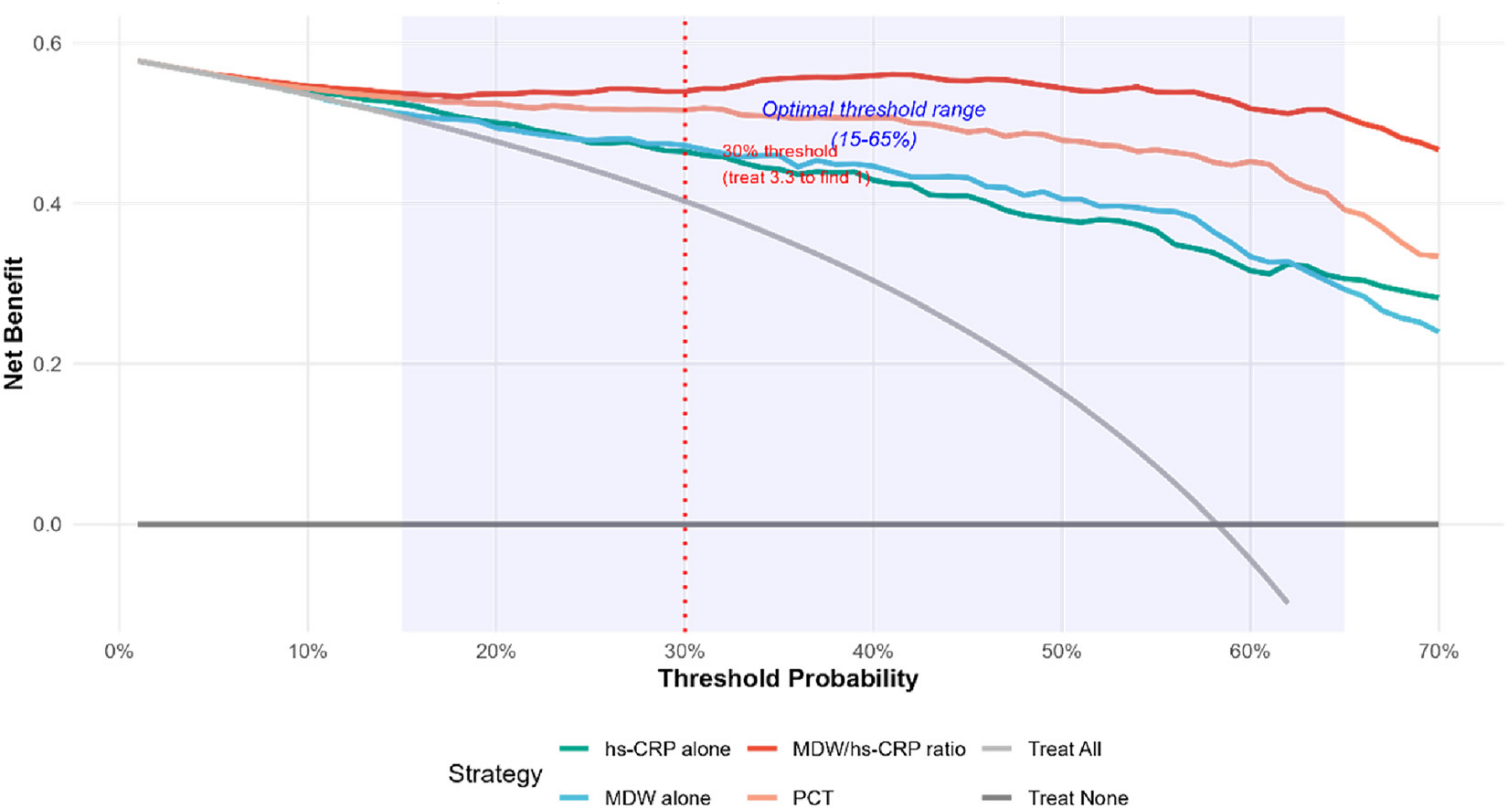
Decision curve analysis showing net benefit of MDW/hs-CRP ratio-guided treatment vs. treating all, treating none, and individual biomarker strategies across threshold probabilities 0–100 %.

Decision curve analysis revealed substantial incremental net clinical benefit from MDW/hs-CRP ratio-guided management compared to alternative strategies across a wide range of threshold probabilities (15–65 %) (Figure 6). At a clinically relevant threshold probability of 30% – representing willingness to treat approximately 3.3 patients to correctly identify one true bacterial CAP case – the ratio-guided approach delivered a net benefit of 0.42. This substantially exceeded alternative approaches: 0.31 for hs-CRP alone, 0.28 for MDW alone, and merely 0.18 for the empiric “treat all” strategy. Translating to absolute terms, ratio-guided management avoided 28 unnecessary antibiotic courses per 100 evaluated patients while preserving 85 % sensitivity for bacterial pathogen detection.

### Clinical outcomes and management impact

The clinical implications of accurate pathogen classification were substantial. Patients correctly classified as bacterial CAP using the MDW/hs-CRP ratio received pathogen-appropriate antibiotic therapy significantly earlier (median time to definitive therapy: 8.2 vs. 14.6 h for misclassified patients, p<0.001) and experienced meaningfully shorter hospitalizations (median length of stay: 5.8 vs. 7.2 days, p=0.008). Conversely, among patients with viral CAP who were correctly identified by the ratio, unnecessary antibiotic exposure was dramatically reduced by 42 % (median duration: 3.2 vs. 5.5 days, p<0.001), achieved without any compromise in clinical outcomes or increased risk of adverse events.

## Discussion

This retrospective study presents the first comprehensive evaluation of the MDW/hs-CRP ratio as a diagnostic biomarker for differentiating pathogen types in CAP, demonstrating superior performance compared to established individual markers. The ratio achieved an AUC of 0.893 for distinguishing bacterial from viral pneumonia, with robust performance across diverse patient subgroups and clinical scenarios. These findings address a critical unmet need in emergency medicine, where rapid pathogen differentiation could substantially improve antimicrobial stewardship and patient outcomes [14].

The biological plausibility of enhanced diagnostic accuracy through MDW/hs-CRP combination reflects distinct but complementary immunological responses to bacterial vs. viral pathogens. Bacterial infections trigger robust monocyte activation with morphological heterogeneity captured by MDW, while simultaneously inducing strong hepatic acute-phase responses reflected in hs-CRP elevation [15]. Conversely, viral infections typically elicit more modest monocyte changes and variable CRP responses, creating differential ratio patterns [16]. This mechanistic framework aligns with recent transcriptomic studies identifying distinct host response signatures between bacterial and viral respiratory infections [17].

Our findings extend previous research on MDW in infectious diseases, which reported AUCs of 0.74–0.94 for sepsis detection depending on clinical setting and reference standards [18]. The superior performance of the ratio format likely stems from normalization of inter-individual variation and amplification of pathogen-specific signals. Similar enhancement has been observed with NLR, where combining neutrophil and lymphocyte counts improved prognostic accuracy in CAP compared to individual cell counts [19]. However, the MDW/hs-CRP ratio demonstrated higher diagnostic accuracy than NLR in our cohort (AUC 0.893 vs. 0.826), possibly reflecting more specific capture of pathogen-associated inflammatory patterns.

Comparison with procalcitonin, currently considered the reference biomarker for bacterial pneumonia differentiation, revealed modest but significant superiority of the MDW/hs-CRP ratio (AUC 0.893 vs. 0.842, p=0.018) [20]. This improvement, while statistically significant, requires contextualization within practical considerations. PCT measurement typically requires specialized immunoassays with longer turnaround times and higher costs, whereas MDW is automatically generated during routine complete blood counts, and hs-CRP utilizes widely available platforms [21]. The combination of superior performance and practical advantages positions the MDW/hs-CRP ratio as an attractive alternative for resource-conscious healthcare systems.

Decision curve analysis provides crucial insight into clinical utility beyond traditional accuracy metrics. The ratio demonstrated net benefit across threshold probabilities of 15–65 %, encompassing the range relevant to most clinical decision-making in CAP [22]. At a 30 % threshold probability, representing moderate clinical suspicion warranting empirical antibiotics, the ratio would correctly guide treatment in an additional 14 patients per 100 tested compared to clinical judgment alone. This translates to meaningful reductions in inappropriate antibiotic exposure without compromising bacterial pneumonia detection.

The consistent performance across subgroups addresses concerns about biomarker generalizability in heterogeneous emergency department populations. Elderly patients, who comprise the majority of CAP hospitalizations and present diagnostic challenges due to atypical presentations, showed preserved ratio performance (AUC 0.882) [23]. Similarly, patients with high comorbidity burden, in whom baseline inflammatory markers may be altered, maintained diagnostic accuracy. The robustness across symptom duration suggests utility throughout the typical presentation window, unlike some biomarkers showing time-dependent variation [24].

Clinical implementation of the MDW/hs-CRP ratio could follow a pragmatic algorithm integrated into existing emergency department workflows. For patients with radiologically confirmed CAP, immediate calculation of the ratio from admission blood work would stratify pathogen probability. Ratios below 0.185 would support bacterial etiology and prompt empirical antibiotic initiation, while higher values would suggest viral etiology, potentially justifying antibiotic withholding pending further evaluation. Alternative thresholds (0.265 for high sensitivity or 0.128 for high specificity) could be applied based on institutional preferences and clinical context. This approach aligns with antimicrobial stewardship principles while maintaining safety through high sensitivity for bacterial detection [25].

### Important limitations and single-center considerations

This study has several critical limitations that warrant careful consideration. Most importantly, the single-center retrospective design substantially limits generalizability of our findings. As all data were collected from a single urban tertiary care emergency department in China, the results may not extrapolate to other geographic regions, healthcare systems, or patient populations. Variations in local pathogen prevalence, antimicrobial resistance patterns, patient demographics, and clinical practice patterns could significantly affect the diagnostic performance of the MDW/hs-CRP ratio. The seasonal distribution of viral pathogens observed in our cohort (68 % occurring between November 2023 and March 2024) may differ in other climates and geographic locations. Additionally, our urban academic center may attract patients with different disease severity and comorbidity profiles compared to community hospitals or rural settings. Therefore, multicenter validation studies across diverse geographic regions, healthcare settings, and seasonal patterns are absolutely essential before the MDW/hs-CRP ratio can be recommended for widespread clinical implementation.

The exclusion criteria applied in this study, while necessary for initial validation, introduce important selection bias that limits applicability to broader emergency department populations. Patients with recent hospitalization, long-term care facility residence, immunosuppression, or prior antibiotic exposure represent substantial proportions of real-world CAP presentations where accurate pathogen differentiation is particularly challenging and clinically important. The restrictive exclusion criteria resulted in 45.5 % of screened patients being excluded, suggesting our findings may primarily apply to community-dwelling, immunocompetent patients presenting early in their illness course without prior treatment attempts. This selective population may have more clear-cut pathogen-specific biomarker responses compared to complex patients with altered immune responses or partially treated infections.

Microbiological diagnosis limitations also warrant emphasis. Despite comprehensive testing protocols, 16.2 % of screened patients with complete biomarker data lacked definitive etiological diagnosis. Among bacterial CAP cases, only 44.9 % were culture-confirmed, with the remainder diagnosed by urinary antigen alone (33.8 %) or PCR methods (21.2 %). These non-culture methods, while clinically practical, may have different specificities compared to traditional culture. The hierarchical diagnostic algorithm, while standardized, may have misclassified some cases, particularly in distinguishing colonization from infection or in mixed pathogen scenarios. Additionally, the possibility of undetected atypical pathogens or emerging respiratory viruses not included in our multiplex PCR panel cannot be excluded.

Critical technical and pre-analytical considerations for MDW measurement require expanded discussion. MDW values are highly dependent on pre-analytical factors including sample handling time, storage conditions, and anticoagulant type. While our protocol specified K3-EDTA tubes with analysis within 2 h, these conditions may not be achievable in all clinical settings. Studies have shown MDW stability up to 4 h at room temperature, but longer delays, temperature fluctuations, or alternative anticoagulants could affect measurement accuracy and consequently the MDW/hs-CRP ratio performance. Furthermore, MDW measurement technology is not standardized across different hematology analyzer platforms and manufacturers [26], 27]. Our study utilized the Beckman Coulter DxH900 analyzer, but other platforms (Sysmex, Abbott, Siemens) may employ different VCS technology parameters and calculation algorithms, potentially yielding different absolute MDW values. The lack of inter-platform comparability studies represents a significant barrier to establishing universal cutoff thresholds. Clinical laboratories would need platform-specific validation and possibly adjusted cutoffs before implementing the MDW/hs-CRP ratio. This technical variability contrasts with more standardized assays like hs-CRP and represents a major practical limitation for widespread adoption.

The optimal cutoff of 0.185 identified in this study requires prospective validation before clinical application, as data-driven thresholds derived from the same dataset used for evaluation typically show optimistically biased performance. External validation in independent cohorts is essential to establish true diagnostic accuracy and appropriate cutoff values. The alternative thresholds provided (0.265 for 95 % sensitivity, 0.128 for 95 % specificity) similarly require prospective confirmation. Additionally, cutoff values may need adjustment based on local pathogen prevalence, seasonal variations, and specific clinical contexts.

The exclusion of immunocompromised patients, while necessary for initial validation, limits applicability to this vulnerable population where pathogen differentiation is particularly challenging and where inappropriate antimicrobial therapy carries highest risks. Future studies should specifically evaluate the MDW/hs-CRP ratio in immunocompromised hosts, including HIV/AIDS patients, solid organ transplant recipients, and those receiving immunosuppressive therapies, with possible development of modified thresholds for these populations [28], 29].

The retrospective design introduces inherent limitations including potential selection bias, information bias, and inability to control for temporal trends or practice pattern changes during the study period. While we attempted to minimize these biases through consecutive patient inclusion and standardized data extraction, prospective validation with predefined protocols would provide more robust evidence.

### Study strengths and future directions

Despite these limitations, our study has several important strengths. The relatively large sample size (n=486) with comprehensive microbiological testing and well-characterized pathogen groups provides robust statistical power for primary analyses. The inclusion of diverse pathogen types (bacterial, viral, atypical, and mixed infections) with species-level identification enhances clinical relevance. Rigorous statistical methodology including DCA, extensive subgroup analyses, and multiple sensitivity analyses strengthens confidence in our findings. The use of established biomarkers as comparators (PCT, NLR) provides meaningful clinical context.

Future research should pursue several important directions. Multicenter prospective validation studies across diverse geographic regions, healthcare settings, and patient populations are the highest priority to establish external validity and generalizability. These studies should include systematic evaluation of inter-platform comparability for MDW measurement across different hematology analyzer systems, with establishment of platform-specific or standardized cutoffs. Investigation in specific high-risk populations currently excluded from our study, including immunocompromised hosts, nursing home residents, and patients with prior antibiotic exposure, would expand clinical applicability.

Integration of the MDW/hs-CRP ratio with clinical prediction rules and other diagnostic modalities through machine learning or artificial intelligence approaches could potentially enhance overall diagnostic accuracy beyond single biomarkers. Serial ratio measurements during treatment could provide prognostic information and guide antibiotic duration or de-escalation decisions, complementing the initial diagnostic utility, similar to demonstrated benefits of MDW kinetic monitoring for mortality prediction in critically ill patients [30]. Pediatric validation studies would address an important knowledge gap, as CAP pathogen differentiation in children presents distinct challenges. Economic analyses comparing MDW/hs-CRP ratio-guided care to standard practice or PCT-guided algorithms would inform implementation decisions in resource-limited settings. Finally, implementation science studies evaluating optimal strategies for incorporating the ratio into clinical workflows, physician acceptance, and impact on actual antibiotic prescribing patterns would be essential for translating research findings into clinical practice improvements.

## Conclusions

This retrospective single-center study demonstrates that the MDW/hs-CRP ratio represents a promising diagnostic tool for rapid differentiation of bacterial from viral CAP in emergency settings, achieving superior performance compared to individual biomarkers and comparable accuracy to more complex assays. With automated availability from routine blood tests, minimal additional cost, and robust performance across patient subgroups within our cohort, this ratio could potentially improve antimicrobial stewardship while maintaining patient safety. However, the single-center retrospective design and technical considerations regarding MDW measurement standardization represent critical limitations that must be addressed. Prospective multicenter validation studies with inter-platform comparability assessments are absolutely essential to confirm these findings and establish implementation strategies for routine clinical practice before the MDW/hs-CRP ratio can be recommended for widespread clinical use.
